# Two Cases of Intubation of the Larynx for Diphtheric Croup

**Published:** 1886-10

**Authors:** Henry D. Ingraham

**Affiliations:** Professor of Diseases of Women and Children, Medical Department Niagara University, Physician to Buffalo German Roman Catholic Orphan Asylum; 405 Franklin Street


### Clinical Reports.

### *TWO CASES OF INTUBATION OF THE LARYNX FOR DIPHTHERIC CROUP.*

BY HENRY D. INGRAHAM, M. D.

Professor of Diseases of Women and Children, Medical Department Niagara University, Physician to Buffalo German Roman Catholic Orphan Asylum.

Sunday morning, Sept. 21st, I was called in consultation by Dr. Banta, to see Gracie O'N——, five years and eight months old. Patient had been suffering from diphtheric croup for several days. The night previous it was thought that she would not live until morning. When we entered the room the little sufferer seemed to be already in death's grasp. Her respirations were 60 a minute, eyes listless, pupils dilated, face livid, extremities cold. It was a fearful struggle for life and it was evident that it could not last long. With the assistance of Dr. Banta and medical students, Banta and Buswell, I succeeded after some little difficulty in introducing one of Dr. O'Dwyer's tubes into the larynx. It gave immediate relief, acted like magic. In a practice of twenty years, I have never seen such prompt and complete relief afforded a patient as the tube gave this little sufferer.

In two minutes the respirations sank to twenty-four a minute, the eyes assumed their natural expression, and the face lost its livid hue. The patient said of her own accord that she felt a great deal better. She complained of the silk thread attached to the tube and asked to have it removed, which was done. After being put into bed she slept quietly most of the time while I remained, about an hour. Her mother said before I left that if she were to die then, the relief had been so great she was glad the tube had been inserted.

Sunday 7 P. M.—Patient comfortable and quiet. Had slept considerable of the time since the insertion of the tube.

Monday Sept. 20th, 10 A. M.—Patient comparitively comfortable, respirations 24 a minute; pulse 115; temperature in axilla, 100½. Was given hydrag. chlo. mit., gr. ss. every two hours. She has taken ice freely, also a little beef-steak and corn starch. No liquids are allowed, at least she is ordered not to have any.

Sept. 21st, 10 A. M.—Patient restless through the night, respirations nearly normal in frequency; pulse 120; temperature 101. Broncho-vescular murmur over both lungs. Has more anxious expression of countenance than yesterday and recovery seems doubtful. Is given :

℞ Tinct. Nux. Vomicæ, - - gtt. xvj.

Ammonii Carb., - - - gr. xvj.

Potass. Acetatis, - - - ʒj.

Syr. Ipecac., - - - - ʒiij.

Syr. Glycyrrhizæ, - - q. s. ad ℥ij.

M. Sig. A teaspoonful every hour.

Strict orders given the mother to give it in two doses, that is give one half of a teaspoonful immediately following the other. Medicine to be given with child lying on her side and no other liquids allowed.

6 P. M.—Patient much better in every respect.

Sept. 22d, 9 A. M.—Three days after the introduction of the tube, patient doing well and indications favorable for her recovery.

CASE II.—Tuesday morning, Sept. 21st:—Called by Dr. Banta to see Eddie B—— nearly four years old. Child has been suffering from diphtheric croup three days, but has been treated by his parents, Dr. Banta not being called until the evening before. Although this child had been sick for a shorter time than Case I. yet the severity of its symptoms are fully as great. I introduced a tube into the larynx but it was soon coughed up. Dr. Banta introduced it again and it was retained for three hours when it was again coughed up. Although the relief in this case was great, yet it was not as marked as in the first case. The parents of the child could see improvement enough, so that they were very anxious to have the tube introduced again after it was coughed up the second time. It was nearly two hours before Dr. Banta and I reached the patient and introduced the tube the third time. About 11 P. M., I was again called to introduce the tube the child having coughed it up a short time before. Dr. Banta being out, my student Mr. Buswell and I introduced it for the fourth time.

At this writing 9 A. M., of the 22d, the tube is retained and the child is doing well. The reason the tube has been coughed up so often in this case is that it is too small. The child needs the same sized tube that Case I. has, and we have only one of that size.

The treatment in the two cases is nearly the same. Dr. W. D. Green and medical student Banta were present when the tube was first introduced in this case. It is too soon to tell what the result will be in these two cases, but all who have seen them believe that by intubation of the larynx a greater degree of relief is more speedily afforded than by any other means, and that the operation is clearly indicated in this class of cases, even if it does nothing more than relieve the terrible suffering of the patient.

So far as I know the greatest objection to intubation is the difficulty in introducing the tube.

I believe these two cases are the first in this city. The result will be given in the next issue of the JOURNAL.

405 Franklin Street.

